# Sources of black carbon to the Himalayan–Tibetan Plateau glaciers

**DOI:** 10.1038/ncomms12574

**Published:** 2016-08-23

**Authors:** Chaoliu Li, Carme Bosch, Shichang Kang, August Andersson, Pengfei Chen, Qianggong Zhang, Zhiyuan Cong, Bing Chen, Dahe Qin, Örjan Gustafsson

**Affiliations:** 1 Key Laboratory of Tibetan Environment Changes and Land Surface Processes, Institute of Tibetan Plateau Research, Chinese Academy of Sciences (CAS), Beijing 100101, China; 2 CAS Center for Excellence in Tibetan Plateau Earth Sciences, Beijing 100101, China; 3 Department of Environmental Science and Analytical Chemistry; The Bolin Centre for Climate Research, Stockholm University, Stockholm 10691, Sweden; 4 State Key Laboratory of Cryosphere Science, Cold and Arid Regions Environmental and Engineering Research Institute, CAS, Lanzhou 730000, China; 5 University of CAS, Beijing 100049, China; 6 Environmental Research Institute, School of Environmental Science and Engineering, Shandong University, Jinan 250100, China

## Abstract

Combustion-derived black carbon (BC) aerosols accelerate glacier melting in the Himalayas and in Tibet (the Third Pole (TP)), thereby limiting the sustainable freshwater supplies for billions of people. However, the sources of BC reaching the TP remain uncertain, hindering both process understanding and efficient mitigation. Here we present the source-diagnostic Δ^14^C/δ^13^C compositions of BC isolated from aerosol and snowpit samples in the TP. For the Himalayas, we found equal contributions from fossil fuel (46±11%) and biomass (54±11%) combustion, consistent with BC source fingerprints from the Indo-Gangetic Plain, whereas BC in the remote northern TP predominantly derives from fossil fuel combustion (66±16%), consistent with Chinese sources. The fossil fuel contributions to BC in the snowpits of the inner TP are lower (30±10%), implying contributions from internal Tibetan sources (for example, yak dung combustion). Constraints on BC sources facilitate improved modelling of climatic patterns, hydrological effects and provide guidance for effective mitigation actions.

## Abstract (web summary)

Black carbon accelerates melting of glaciers in the Himalayas and Tibet, yet the source of these aerosols remains enigmatic. Here, the authors use isotope fingerprinting techniques to determine the origin of black carbon preserved in glacier ice cores recovered from the Himalayas and Tibetan Plateau.

## Graphical abstract

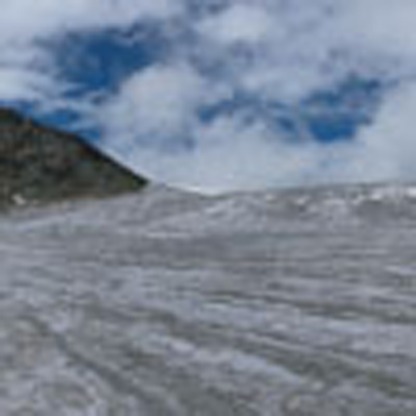

The Himalaya-Hindu-Kush and Tibetan Plateau, which are collectively referred to as the Third Pole (TP), contain the largest ice mass on the planet outside of the polar regions. Unlike the Arctic and Antarctic regions, the TP is situated in a mid-latitude location and is located in the immediate vicinity of densely populated and industrialized regions. Of particular importance is its proximity to two countries (China and India) that emit the most climate-warming black carbon (BC) aerosols^1^,^2^,^3^,^4^ (Fig. 1). Therefore, the TP is considered to be one of the most vulnerable regions to effects of BC aerosols. The long-range transport to and deposition of BC aerosols in the TP is attracting considerable attention because of BC's effects on the transformation of hydrological and radiative forcing in the East and South Asian regions^5^,^6^,^7^,^8^. In addition, BC aerosols are believed to play a considerable role in the melting of mid-latitude glaciers because of both the heating of aloft air masses transported into the TP and the albedo effects of deposited BC^9^,^10^,^11^,^12^,^13^.

Studies based on global chemical-transport models have yielded varying predictions of source region contributions, suggesting that BC deposited on TP glaciers stems from heavily polluted regions on the Indian subcontinent and/or from East Asia^4^,^14^,^15^,^16^,^17^. However, these models are uncertain with respect to source-area emissions and air transport patterns across the highly elevated topographical mountain features of the TP. Thus, direct observational constraints are urgently needed to reduce uncertainties regarding sources of BC reaching the TP^18^,^19^. Because of the TP's overall inaccessibility, *in situ* BC aerosol observations for the region are lacking^10^,^19^,^20^,^21^, as are data on carbon isotopic compositions, which have recently been proven useful for quantitatively constraining sources of BC^20^,^21^,^22^,^23^,^24^,^25^,^26^. Furthermore, the limited comparisons of model-simulated and measured BC concentrations in the TP are not in good agreement^4^,^27^; these differences may be attributable to several causes, including uncertain emissions inventories, the complicated effects of topographic transport steering^2^, and source-related differences in the scavenging of different BC source types^28^,^29^.

Emissions inventories are severely challenged in terms of particle emissions from incomplete combustion (for example, BC), especially for sources characterized by poor levels of combustion efficiency. Such sources are prevalent in South and East Asia and include the open combustion of garbage and crop residues, the use of brick kilns and household cooking/heating with firewood, and the burning of animal dung and coal briquettes. Studies of BC emissions inventories for Asia have reported uncertainties as high as factors of 2–4 (refs 2, 30). The Δ^14^C/δ^13^C-based isotopic source-diagnostics of ambient BC aerosols demonstrate that emissions inventory models systematically under-predict fossil fuel-sourced BC contributions relative to biomass-sourced BC fractions for both India^22^ and China^21^,^31^. Because BC emissions from fossil vs biomass combustion have different properties^32^, improved source discrimination methods may help increase the understanding of both radiative and transport processes^33^.

Fossil fuel-sourced BC appears to have approximately twice the particle-specific warming potential of biomass-sourced BC^2^,^32^. One major challenge facing the successful modelling of BC transport in mountain regions is the updraft transport from source regions to the TP^2^; during this process, the scavenging of aerosols on slopes remains unknown, particularly for biomass vs fossil fuel BC.

To address these uncertainties, the dual-carbon-isotope (Δ^14^C and δ^13^C) fingerprint of BC, which is diagnostic for fossil fuel vs biomass combustion and for source regions, was investigated using aerosol and glacier snowpit samples collected from the hitherto most extensive geographical collection of BC observations on the southern, central and northern mountain chains of the TP (Fig. 1).

The natural abundance radiocarbon signal (Δ^14^C) of BC in the present-day atmosphere has proven to be effective in quantitatively apportioning the relative contributions of fossil fuel vs biomass combustion^21^,^22^,^34^. The clearly different Δ^14^C-BC source fingerprints of South Asia (50±8% fossil)^20^,^22^ vs East Asia (80±6% fossil)^21^,^31^ allows the relative influences of BC from these two regions to the TP to be differentiated. An even higher resolution in BC source apportionment was recently achieved by characterizing the dual Δ^14^C/δ^13^C-BC fingerprint, which allowed the separation of the relative contributions from liquid fossil fuel, coal and biomass burning to severe haze events in China^31^. Thus, applying the Δ^14^C/δ^13^C-BC across the TP should facilitate identifying the sources of the BC that is melting the glaciers and from what process and where this BC originates.

In the present study, both air particles and particles deposited on glaciers were collected from multiple sites in the TP. From 2013 to 2014, bulk aerosols were collected from nine stations across two Himalayan valleys (Mustang and Langtang) and in the southern TP (Fig. 1 and Supplementary Table 1). Furthermore, snowpit samples from eight glaciers were obtained from a region spanning from the Himalayas in the south to the Qilian Mountains in the northeastern TP (Supplementary Table 2). The elemental carbon (EC) component, which is the common chemical/mass definition of BC, was isolated from each sample via thermal-optical analysis, and evolved CO_2_ was purified online and collected using a cryo-trap^21^,^31^. The combined Δ^14^C-δ^13^C fingerprint of BC was used to identify the sources of BC to the Himalaya-Tibetan Plateau with respect to both source regions and the relative importance of different combustion source classes. We found approximately equal contributions from biomass burning and fossil fuel combustion to the Himalayas. Although the isotopic fingerprint is largely consistent with the Indo-Gangetic Plain (IGP) being a main source region for the Himalayas, the more ^14^C-depleted signal in the northern Tibetan Plateau corresponds to a Chinese source signature. Furthermore, the relatively contemporary ^14^C signal of the central regions of the TP reflects less fossil fuel use than the South Asian and East Asian fingerprints, suggesting a putative internal/domestic source putatively resulting from the combustion of yak dung by local residents.

## Results

### Δ^14^C of BC aerosols across the Himalayas

The air mass transport of BC from the heavily polluted IGP region to the Himalayas has been proposed to follow specific pathways. In particular, south-north-trending valleys, such as the Mustang and Langtang Valleys, may play important roles^35^. In the present study, we observed significantly decreasing atmospheric BC concentration gradients in these two valleys (Supplementary Fig. 2), suggesting BC deposition and dilution as the air moves north and upward along them (Fig. 2a). Intriguingly, current ^14^C-BC signals show a consistent trend of changing BC source signatures along both valleys, with fossil fuel BC contributions (*f*_*fossil*_) ranging from 70±11 to 58±3% and from 49±7 to 23% along the south-north gradients in the Langtang and Mustang Valleys, respectively (Fig. 2a). The high *f*_fossil_ value in the southern Langtang Valley reflects urban effects from Kathmandu, whereas those found for other southern sites are in strong agreement with the *f*_fossil_ of BC for the IGP of northern India^22^. Because fossil fuel combustion-sourced BC is at least as likely to be transported over long distances, decreasing *f*_fossil_ values moving up valleys and into the TP suggest a significant influence of local biomass-burning activities on the total BC load of the TP.

Another mechanism of pollutant transport into the TP from the IGP involves movement through an aloft conveyor, which gives rise to high levels at elevated tropospheric altitudes^36^,^37^. Although the BC aerosol concentration at Namco is very low (0.09±0.02 μg m^−3^), its fossil fuel contribution (46±6%) falls within the range found in the atmosphere of the IGP^22^,^31^ (Fig. 2a), suggesting that Namco receives predominantly long-range-transported pollutants from outside of the TP. These isotope-based source apportionment results confirm previous suggestions that BC aerosols from the IGP can be transported to the Himalayas^38^,^39^,^40^ and even further to the southern Tibetan Plateau^19^,^41^. The city of Lhasa, which is located in the southern TP, exhibits a relatively depleted ^14^C-BC signal (that is, reflecting a larger fossil fuel contribution) and is instead similar to that commonly found in East Asia (Supplementary Table 4). We suggest that the fuel structure of Lhasa is similar to that of urban areas across China^21^,^42^,^43^,^44^.

This study also assess seasonal variations in atmospheric BC concentrations and their relative source contributions in the TP because such variations are important inputs for radiative forcing assessments. The BC concentrations decrease during the monsoon period in the Himalayas (Supplementary Fig. 3), presumably because of heavy precipitation^38^,^45^. In this study, isotope-based source apportionment results further suggest a relative increase in fossil fuel BC at all of the examined stations (Supplementary Fig. 3). This is likely attributable to more efficient scavenging of biomass-sourced BC from the atmosphere via precipitation, as observed in South Asia^46^ and Europe^47^, and/or lower levels of biomass fuel consumed by local TP residents during the warmer monsoon period. East Asia may also represent a possible source during the monsoon period^17^,^48^, contributing to the slightly elevated *f*_fossil_ values associated with this period. Consequently, fossil fuel-sourced BC plays a relatively more important role in climate forcing in the TP during the monsoon period. Because this temporal phenomenon was observed in both urban and remote areas, it may represent a general feature that could also apply to other Asian regions.

### Δ^14^C of glacier snowpit BC across the TP

**Figure 1:**
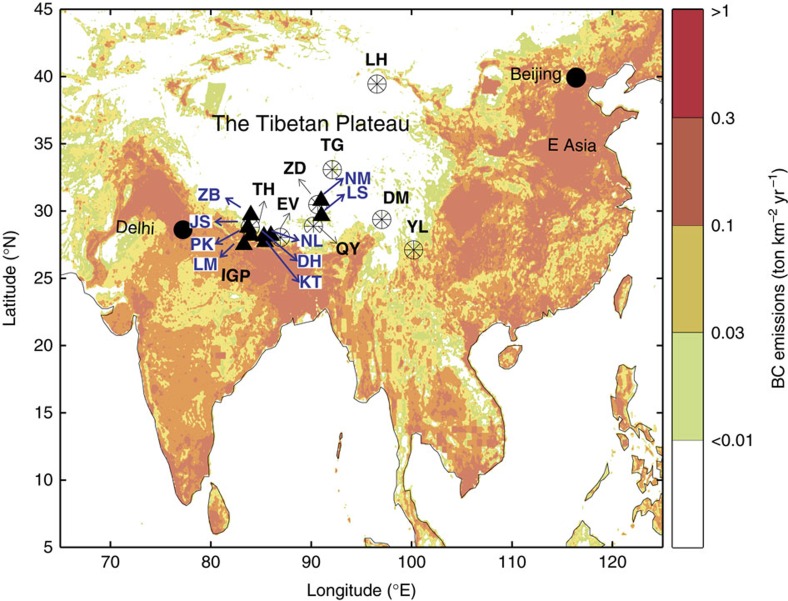
Map of sampling sites. The background image presents the model-predicted BC emission inventories for South and East Asia created using MATLAB (version 2014b) based on data drawn from http://inventory.pku.edu.cn/download/download.html^66^. Triangles denote aerosol sampling sites, and circles denote snowpit sampling sites. NM, LS, NL, DH, KT, ZB, JS, PK and LM indicate the following aerosol stations: Namco, Lhasa, Nyalam, Dhunche, Kathmandu, Zhongba, Jomsom, Pokhara and Lumbini, respectively. LH, TG, ZD, YL, DM, QY, EV and TH represent the following glaciers: Laohugou No. 12, Xiaodongkemadi, Zhadang, Yulong, Demula, Qiangyong, East Rongbuk and Thorung, respectively.

**Figure 2:**
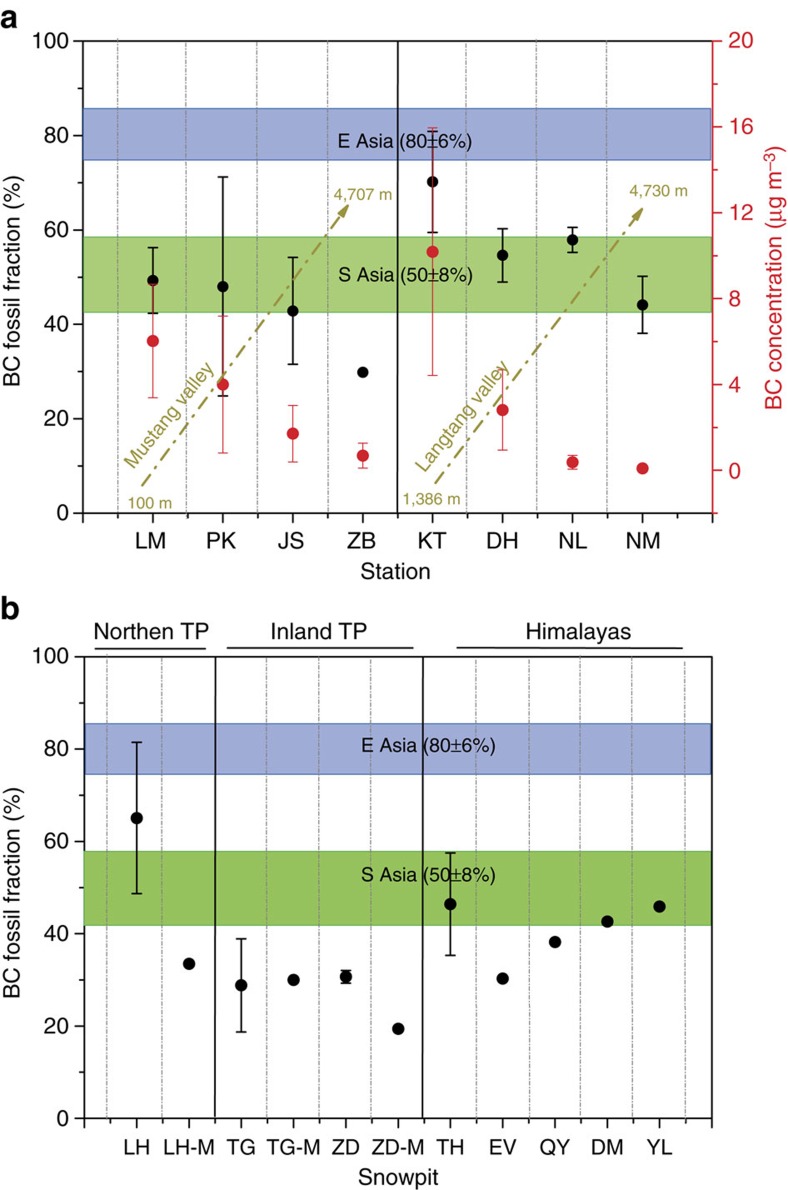
Fossil fuel contributions of black carbon in the studied samples. (**a**) Aerosol samples collected from two valleys across the Himalayas. (**b**) Snowpit and snow samples collected during the monsoon period (LH-M, TG-M and ZD-M). For aerosol samples: The error bars (1 s.d.) represent 1–4 samples collected during different seasons for each station (Supplementary Table 4). For the snowpit samples: The error bars (1 s.d.) are based on two replicates for each snowpit (one sample for EV) (Supplementary Table 6). Some bars are so small that they are hidden by symbols. Data for South Asia (solid green band) and North China (solid blue band) were adopted from refs 20, 22 and refs 21, 31, respectively. To investigate the decreasing concentrations and fossil fuel contributions of BC from South Asia to the remote area of the TP, data for the city of Lhasa, which is heavily polluting and has a fuel profile similar to that of most Chinese cities, are excluded. The error bars of the fossil fuel contributions and BC concentrations correspond to 1 s.d. for samples collected on different dates. NM, NL, DH, KT, ZB, JS, PK and LM indicate the following aerosol stations: Namco, Nyalam, Dhunche, Kathmandu, Zhongba, Jomsom, Pokhara and Lumbini, respectively. LH, TG, ZD, YL, DM, QY, EV and TH represent the following glaciers: Laohugou No. 12, Xiaodongkemadi, Zhadang, Yulong, Demula, Qiangyong, East Rongbuk and Thorung, respectively.

**Figure 3:**
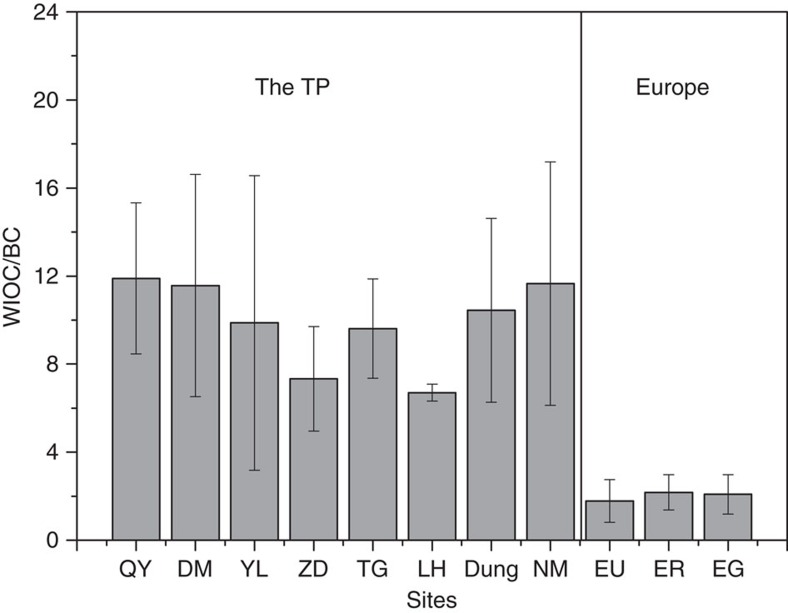
WIOC/BC ratios for snowpit and aerosol samples from the Third Pole and Europe. The error bars represent 1 s.d. of the WIOC/BC ratios for each station. The sample numbers from QY, DM, YL, ZD, TG, LH, Dung, NM, EU, ER and EG were 9, 7, 6, 5, 8, 3, 9, 8, 7, 12 and 5, respectively. QY, DM, YL, ZD, TG and LH indicate the following glaciers: Qiangyong, Demula, Yulong, Zhadang, Xiaodongkemadi and Laohugou No. 12, respectively. Dung and NM refer to aerosol samples of yak dung combustion and outdoor air from Namco, respectively. EU, ER and EG refer to particles drawn from urban areas^25^, remote areas^47^ and glacial areas across Europe^26^, respectively.

**Figure 4:**
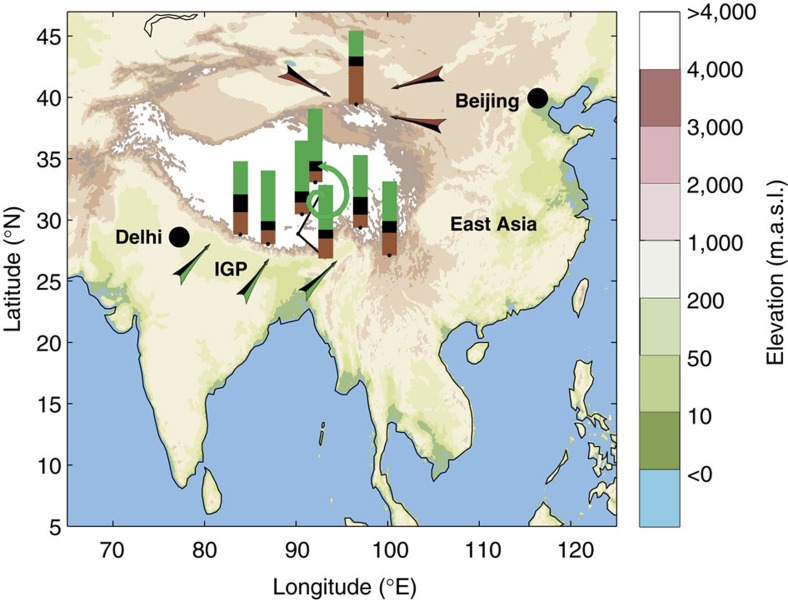
Relative contributions of the combustion of three fuel classes to Third Pole snowpit sample black carbon. Green, black and brown bars represent biomass, liquid fossil fuels and coal combustion, respectively. The source signatures of the ^14^C- and ^13^C of these three fuels are presented as mean±s.d. values in Supplementary Table 7. Black and brown arrows denote the transport of BC from East Asia, black and green arrows indicate the transport of BC from South Asia, and green arrows represent BC emissions from local/domestic activities in the TP.

BC emitted from the IGP and East Asia can be transported to and deposited on glaciers in the TP, especially in its fringe areas. The BC concentrations in snowpit samples collected across the TP (Fig. 1) varied considerably (11–133 ng g^−1^), in agreement with previous results reported for snowpits in the TP, with high concentrations in the northern TP (Supplementary Table 5)^49^. The ^14^C signal of snowpit BC shows clear spatial trends, with increasing levels of ^14^C enrichment (that is, increasing contributions from biomass combustion) from the Himalayas (Thorung (TH)) and northern TP (Laohugou (LH)) to inland Tibet (Tanggula (TG) and Zhadang (ZD)) (Fig. 2b). This source-contribution trend was independent of the BC concentration (Supplementary Fig. 4), demonstrating that ^14^C provides more distinct source-diagnostic information than the concentration and that fossil fuel-sourced BC is transported from outer source regions to central areas in the TP. The fossil fuel contributions of BC found in all of the TP snowpit samples were lower than those found in European Alpine ice cores (83±2%) (ref. 26). The BC from a European glacier was shown to exhibit a high ratio of fossil fuel contributions, with *f*_fossil_ values succeeding those of urban areas in the region; these findings suggested the preferential long-range transport of BC from fossil fuels compared^23^ with that of BC from biomass combustion. The water-insoluble organic carbon (WIOC)/BC values were found to be much higher in the snowpits of the TP glacier than in the European Alps and similar to those of Namco aerosol and biomass-burning-sourced particles (Fig. 3). These findings are consistent with locally sourced biomass combustion particles contributing substantially to the BC loading of TP glacier region (Fig. 4). Taken together, the relatively enriched ^14^C and high WIOC/BC ratios suggest that a large proportion of BC in the TP glaciers is derived from biomass burning. Spatially, the highest fossil fuel contribution (66±16%), which was observed in the remote northernmost snowpit at LH in the northern TP, is similar to those commonly found in the developing areas of East Asia (80±6%) (refs 21, 31) and thus reflects the effects of anthropogenic emissions transported from north-western China to glaciers in the northern TP (Fig. 1). Correspondingly, the fossil fuel BC contributions among glaciers along the southern slopes of the Himalayas (TH) (46±11%) reflect the effects of IGP (50±8%) (refs 20, 22). Hence, source-diagnostic BC observations for TP glaciers in both the northern and southern fringe regions are consistent with BC aerosols originating from East and South Asia, respectively.

We also found that ^14^C is enriched (that is, more contemporary) in snowpit BC in the inland TP (for example, TG and ZD) relative to the levels in the IGP and East Asia (Fig. 2b). This finding may indicate sizeable contributions of BC emissions from local TP residents who largely rely on burning yak dung for daily cooking and heating^50^,^51^,^52^, thereby significantly affecting glacier BC in the inland TP. Consequently, whereas previous research has assumed that the BC in the TP stems mainly from long-range transport processes from either the IGP or East Asia^4^,^15^, the enriched ^14^C in the BC of inland TP glaciers (for example, ZD and TG) found here more likely indicates contributions from local, previously overlooked Tibetan sources. Taken together, the results of this extensive observation-based source-diagnostic study provide strong isotope-based evidence that biomass-sourced BC plays a quantitatively more important role in TP glacier melting than fossil fuel-sourced BC, especially in the inland TP, and presumably arises mainly from domestic sources^17^.

## Discussion

A combined analysis of the δ^13^C and Δ^14^C fingerprints can further refine the source apportionment of BC^20^,^21^,^22^. The contributions of coal-combustion-sourced BC are the most significant for the LH glacier, which is in line with the high coal-consumption patterns characteristic of north-western China (Fig. 4) (ref. 31). Correspondingly, the δ^13^C signal corresponding to liquid fossil fuel is in good agreement with the δ^13^C of BC in glaciers in the Himalayas and inland TP, highlighting the influence of South Asian sources. Taken together, the Δ^14^C/δ^13^C-BC signals further decipher the sources of BC across the TP, revealing an approximately equal influence of biomass combustion sources within the southern TP/Himalayas that mainly stem from emissions from the IGP. We also found that the northern TP is influenced by emissions from north-western China, whereas the inland TP experiences non-negligible effects from internal domestic sources of BC (Fig. 4). These observation-based source apportionments of BC facilitate improved modelling of the climatic and hydrological effects of BC in the TP and guide policy actions aimed to effectively mitigate emissions.

## Methods

### Snowpit sample collection

Eight snowpits were collected from col or firn basins of glaciers in the TP during May and June of 2012–2014 (Fig. 1 and Supplementary Table 2). Snow samples were collected at a vertical resolution of 20–30 cm using a pre-combusted stainless steel shovel (550 °C, 6 h) and were stored in sterile 5-L Whirl-Paks bags (Nasco, Fort Atkinson, WI, USA). The frozen samples were transported back to the laboratory and transferred into polytetrafluoroethylene bags. The samples were allowed to thaw at room temperature and were then subjected to ultrasonic treatment and filtration through a pre-combusted and pre-weighted quartz fibre filter (47 mm in diameter, Whatman Corp) twice. Samples from clean and dirty snow layers were filtered separately, and the filtered particles were freeze-dried and weighed. Subsequently, the filters were folded, wrapped in pre-combusted aluminium foil, packed into airtight plastic bags, and stored at −20 °C for further analysis. The water volume that passed through each filter was recorded. Initial tests showed that the split times between the WIOC and BC values for snowpit samples with heavy particle loads were longer (later), resulting in the determination of lower or even zero BC concentrations because of the difficulties associated with resolving small changes in laser signals under such heavy loading conditions. To mitigate this problem, we sought to maintain a low particle load on the filter to more readily resolve the split time.

Samples from snowpits were collected during the spring, mainly during the non-monsoon period (September–May), rather than all year. This is because snow deposited during the monsoon period occasionally melts because recent dramatic warming patterns^53^,^54^, increased light-absorbing particles in glacier^13^ and decreased precipitation levels^55^. Therefore, fresh snow samples were also collected from the firn basins of three glaciers (ZD, TG and LH) within 12 h of heavy snow events during the monsoon period (Supplementary Table 2). Potential source regions for snow events during the monsoon period were estimated using air mass backward trajectories (http://www.arl.noaa.gov/ready/hysplit4.html). The air masses of these monsoon period snowfall events moved from West China and the Hexi Corridor to the LH glacier (Supplementary Fig. 5a) and from South Asia (Supplementary Fig. 5b,c) to the TG and ZD glaciers, respectively.

### Aerosol sample collection

Total suspended particle (TSP) samples were collected from pre-combusted quartz fibre filters (90 mm in diameter, Whatman Corp.) installed at seven stations along two valleys (the Mustang and Langtang Valleys) across the Himalayas from the IGP to the TP from January of 2013 to December of 2014 using TSP cyclones at a flow rate of 100 l min^−1^ (TH150-A, Wuhan Tianhong INST Group) (Supplementary Table 1). Each sample was collected for 24 h during the non-monsoon season and for 48 h during the monsoon season. The collected samples were kept frozen until analysis, which used the thermal-optical transmittance (TOT) method (detailed below). TSP samples were also collected from the city of Lhasa and from the Namco station between 2013 and 2014. Because of the low BC concentrations at the Namco station, each aerosol sample was collected for ∼20 days using the same TSP cyclone. Although some of the collected thermodynamically unstable organic species may have been chemically transformed by strong oxidants during these long sampling periods, the carbon isotopic compositions of collected recalcitrant BC were expected to remain stable.

### BC measurement

The BC mass contents of the filtered snow and TSP samples were quantified using the TOT technique. Briefly, filters were acidified by fumigation in open glass Petri dishes held in a desiccator with >37% HCl acid for 24 h to remove carbonates and were subsequently dried at 60 °C for 1 h to remove any remaining HCl acid. A 1.5 cm^2^ piece of acid-treated filter was cut out and analysed using a TOT carbon analyser (Sunset Laboratory, Tigard, OR, USA) following the National Institute for Occupational Safety and Health (NIOSH) method 5040 to determine BC and organic carbon (OC) concentrations of the aerosol samples and the WIOC concentrations of the particles from the collected snow sample. Sucrose standard and other reference materials were also subjected to these measurements^21^.

### CO_2_ isolation and Δ^14^C and δ^13^C analysis

The filter area required for the subsequent ^14^C measurements was determined based on the measured BC concentration. The filters were then subjected to the Sunset-NIOSH-5040 protocol, and the CO_2_ produced was cryotrapped during the BC combustion phase after removing the water and sulfur-containing gases online^21^. Purified CO_2_ was then transferred in flame-sealed glass ampules to the United States National Science Foundation, National Ocean Science Accelerator Mass Spectrometry facility at the Woods Hole Oceanographic Institution (Woods Hole, MA, USA). The radiocarbon analysis results are reported as per mil deviations (Δ^14^C) relative to National Bureau of Standards oxalic acid. The parameters of the studied samples are listed in Supplementary Tables 4 and 6. This type of analysis has been used in previous studies on aerosol^21^,^22^,^23^,^25^,^31^ and snow/ice core samples^26^,^56^. Because of the inert (unreactive) nature of BC, minimal isotopic fractionation is expected during its long-range transport^2^,^31^. For snowpit samples, isotopic measurements were performed by assuming that the BC collected by filtration^57^,^58^ (here, 77±17%; *n*=16 (Supplementary Fig. 1)) is representative of the total BC content found in snow. A putative effect of OC charring on the estimated BC Δ^14^C was also considered. The split time between OC and BC was similar for the snowpits and aerosols, suggesting that such effects are limited (Supplementary Table 3), which is consistent with earlier sensitivity test results for charring^21^.

### Isotope mass balance model

A simple binary mixing model was used to obtain the fractional contributions of biomass (*f*_biomass_) and fossil fuels (*f*_fossil_=1−*f*_biomass_) to the carbonaceous aerosol components of the measured samples:


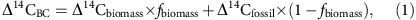


where Δ^14^C_BC_ is the measured radiocarbon content of the BC component, and Δ^14^C_fossil_ is –1,000‰. The Δ^14^C biomass end member falls between +70‰ (freshly produced biomass) and +225‰ (wood). A large portion of the TP is covered by grassland, and yak dung is the main fuel source used by local residents. In contrast, forests are distributed across the southeastern TP, and in this area, wood is the local residents' main fuel source. Therefore, a Δ^14^C_biomass_ end member of +70‰ (ref. 59) was adopted for samples obtained from Namco and all of the snowpits but not for those from DM, YL and TH glaciers and other stations (200‰) in the model calculations^60^. The carbon isotope signatures of BC for the snowpit and aerosol samples were assessed using a statistical source apportionment model to quantify relative contributions from the three major emission source classes: biomass, coal and liquid fossil fuels (for example, petroleum, gasoline and diesel)^31^. The comprehensive uncertainties associated with this approach were assessed using Andersson and colleagues' Bayesian Markov Chain Monte Carlo numerical simulation procedure^31^.

### Assessment of snowpit BC collection efficiency

The BC-collection efficiency of the filtration of melted snow samples was investigated. Adding a coagulant, such as NH_4_H_2_PO_4_, has been shown to increase the BC-collection efficiency of filtration^57^,^58^. Hence, this technique's BC-collection efficiency level was tested by adding (or not adding) NH_4_H_2_PO_4_ to 16 snowpit samples before filtration. More specifically, the melted snow sample was divided into equal parts, NH_4_H_2_PO_4_ (1.5 g per 100 ml of sample) was added to one of the subsamples, and the mixture was then magnetically stirred for 10 min. All subsamples (with and without NH_4_H_2_PO_4_) were then simultaneously filtered through quartz filters for WIOC and BC concentration measuremen^57^. The ratio of the BC contents in samples with and without NH_4_H_2_PO_4_ was determined to be 77±17% (Supplementary Fig. 1), which denotes a fairly high degree of recovery. These values were slightly higher than those found for snow samples collected in Japan (69%) (ref. 57), and they were much higher than those found for precipitation samples (38%) (ref. 58). Hence, the BC collected via the filtration method without NH_4_H_2_PO_4_ in this work corresponds to the majority of the BC in each sample. Achieving such high BC-recovery rates from TP snowpit samples is reasonable. Indeed, the BC grain-size distributions in snow tend to be larger than those in rainwater, resulting in higher BC collection efficiencies for snow samples^61^, as reported for snow samples from Japan^57^. In addition, a previous study suggested that the filter efficiency of quartz filters can be influenced by several factors, such as possible WIOC loading on filters and BC grain sizes^62^. The WIOC/BC ratio of the TP snowpit sample examined here is much higher than those reported for the European Alps^26^ and Japan^57^, and thus, we assumed that heavy WIOC loading on filters increases the BC collection efficiency. Kuchiki *et al.* also suggested that

‘compacted BC aggregates and BC dust aggregates often form in snow samples without a coagulant, suggesting that the larger-size snow impurities in the natural snow samples led to the high collection efficiency of the quartz fibre filter even without a coagulant'^57^. This is likely true for the glacier snowpit we examined, in which we observed some visible particles. Furthermore, previous studies have reported high BC collection efficiencies for glacier samples from the TP^13^ and elsewhere^63^,^64^.

Taken together, these results indicate that although not all BC can be collected from melted snow by filtering directly through quartz filters, high recoveries are possible. Furthermore, making a first-order assumption that any BC loss should proportionally affect BC derived from fossil fuel and biomass burning, resulting in negligible effects on the determined carbon isotopic composition, seems reasonable.

### Potential influence of charring on BC concentrations

The putative effects of the inadvertent inclusion of instrument-induced pyrolysed-C (PyrC from OC) in the ^14^C-EC isolate were considered. The formation and subsequent oxidative removal of PyrC constitute a well-recognized process that is inherent in the design of the Sunset-TOT method. The PyrC is accounted for by monitoring the laser transmission signal through the filter online, and this method is designed to burn off any remaining PyrC during the subsequent oxidation step. However, this process may also introduce a certain degree of carbon mixing from different sources (that is, some ‘true' EC is burned off as PyrC, and some PyrC is included in the observed EC fraction). However, this potential mixing effect is not expected to substantially influence concentration estimates because the mass absorption cross-sections of PyrC and EC are assumed to be similar. Indeed, several sensitivity tests of this hypothetical charring-exchange process have demonstrated that its effect should be minimal^21^,^65^. Furthermore, in this study, the particle load was kept as low as possible, and the WIOC/BC ratios of the snowpit samples were similar to those found in aerosols (Supplementary Table 3 and Fig. 3); both of these points suggest a low risk of charring.

### Data availability

All data will be publicly available in Stockholm University's Bolin Centre Database (http://bolin.su.se/data/).

## Additional information

**How to cite this article:** Li, C. *et al.* Sources of black carbon to the Himalayan–Tibetan Plateau glaciers. *Nat. Commun.* 7:12574 doi: 10.1038/ncomms12574 (2016).

## Supplementary Material

Supplementary Information Supplementary Figures 1-5, Supplementary Tables 1-7 and Supplementary References
