# Supplementary material for: Sources of black carbon to the Himalayan–Tibetan Plateau glaciers
Source: Nat Commun. 2016 Aug 23;7:12574. doi: 10.1038/ncomms12574 (PMC4996979; doi:10.1038/ncomms12574)
Supplement: Supplementary Information — Supplementary Figures 1-5, Supplementary Tables 1-7 and Supplementary References [file ncomms12574-s1.pdf]

Supplementary Information

1. Supplementary Figures

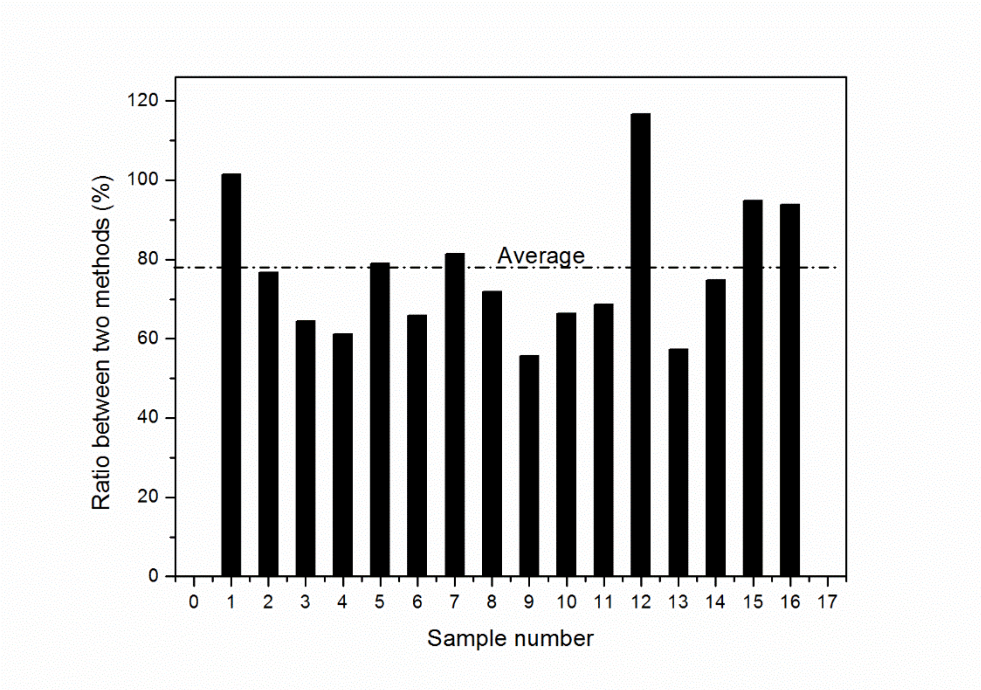

Supplementary Figure 1 | Proportions of BC collected by filtering samples with and without $NH_4H_2PO_4$.

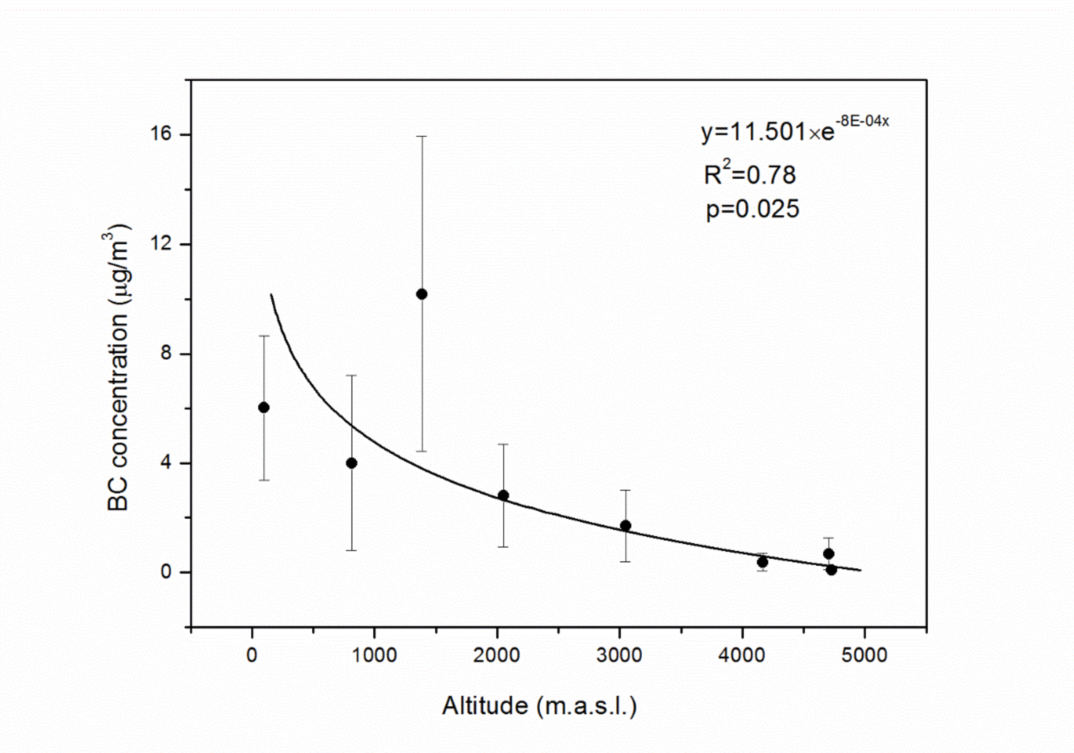

Supplementary Figure 2 | Significantly decreasing BC concentrations across the Himalayas. The error bars represent 1 SD of the BC concentrations for each station.

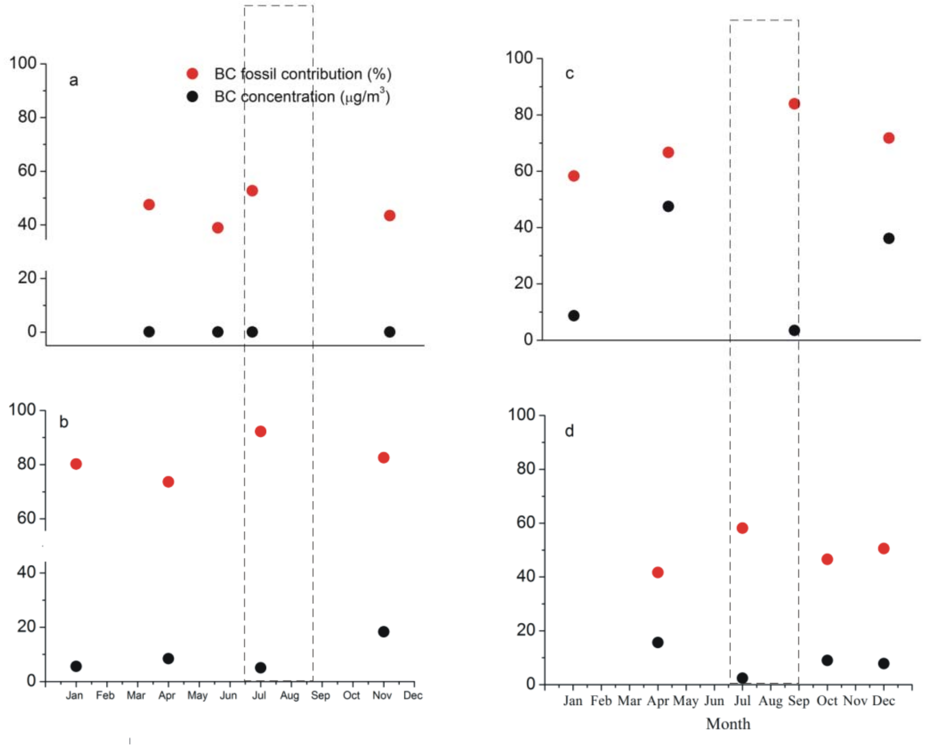

Supplementary Figure 3 | Seasonal variations in the fossil fuel contributions (%) and BC concentrations. (a-d) Namco, Lhasa, Kathmandu and Lumbini, respectively. Other sites are not shown because ^{14}C was only determined in samples collected over fewer than four seasons; samples indicated in boxes were collected during the monsoon period.

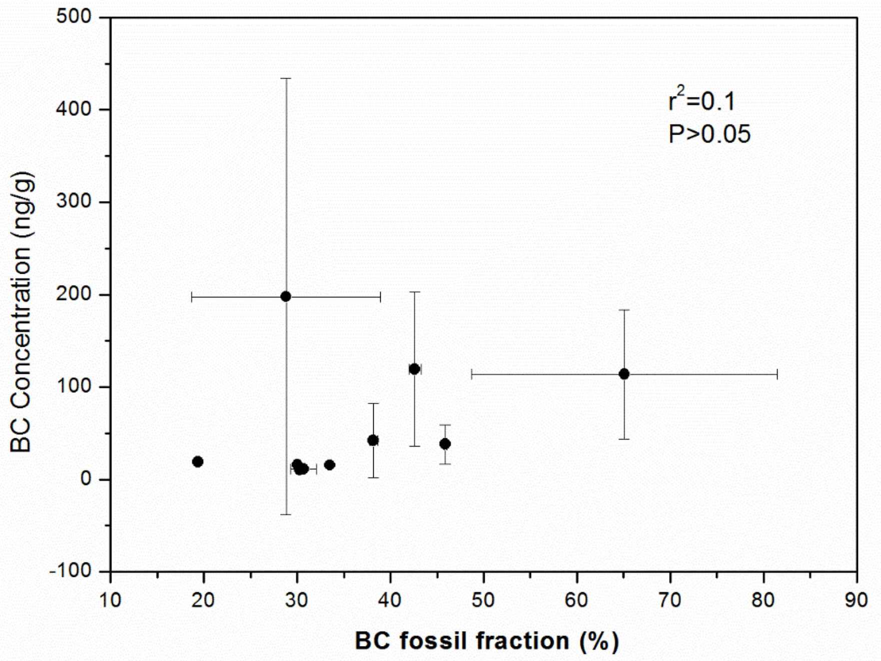

Supplementary Figure 4 | Relationship between f_{fossil} and the BC concentration for all of the studied snow samples. Error bars represent 1 SD of the BC concentrations and fossil fuel contributions of BC (%) for the studied snowpits.

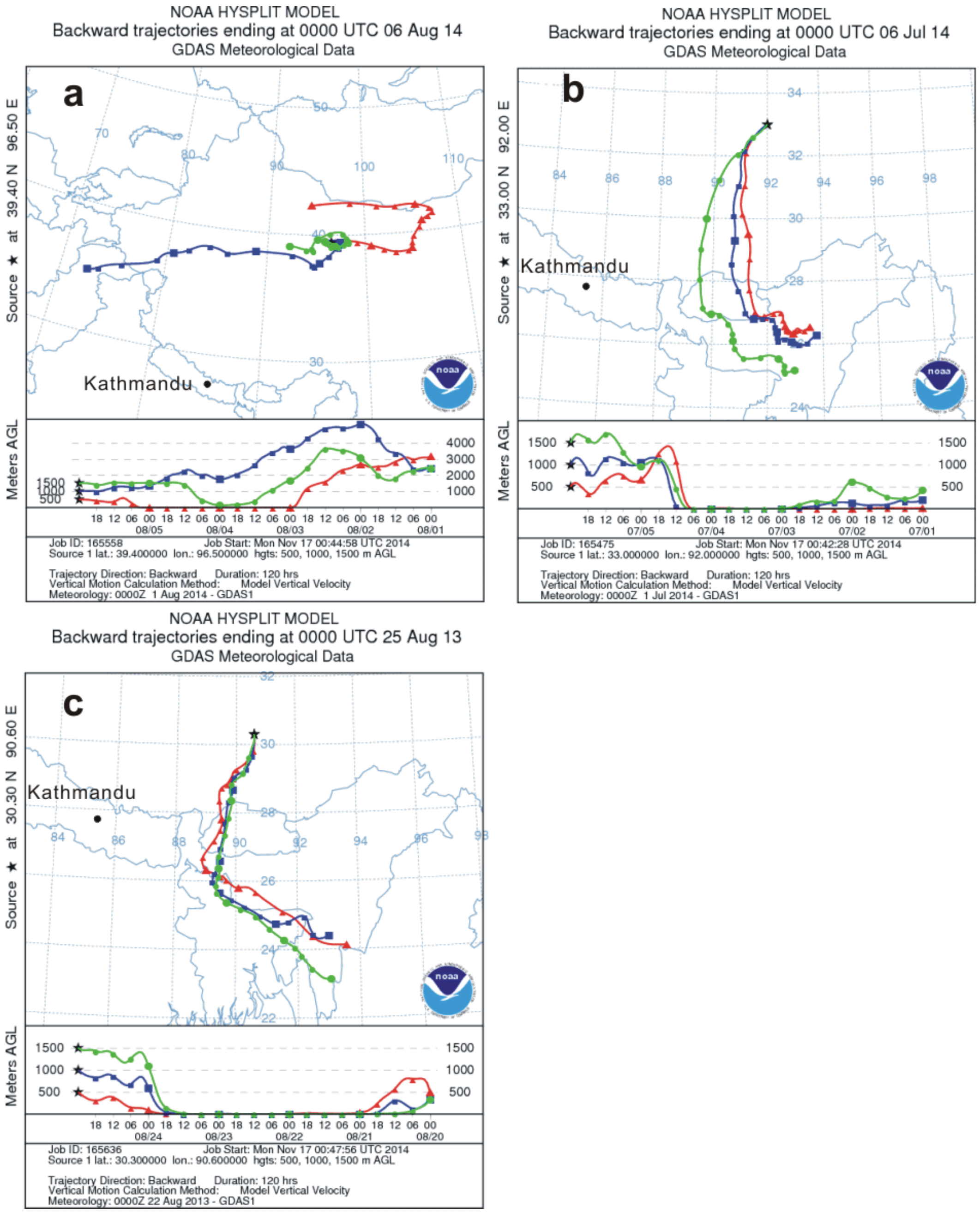

Supplementary Figure 5 | Backward trajectories for monsoon snowfall events on the glaciers. (a-c) the Laohugou No 12, Xiaodongkemadi and Zhadang glaciers, respectively.

Supplementary Tables

Supplementary Table 1 | Locations of stations at which total suspended particle (TSP) was collected.

Station ID	Station	Latitude<br>N	Longitude<br>E	Elevation<br>m a.s.l
NM	Namco	30°46′	90°59′	4730
LS	Lhasa	29°38′	91°01′	3640
NL	Nyalam	28°10′	85°59′	4166
DH	Dhunche	28°07′	85°18′	2051
KT	Kathmandu	27°42′	85°24′	1386
ZB	Zhongba	29°42′	83°59′	4704
JS	Jomsom	28°46′	83°43′	3048
PK	Pokhara	28°11′	83°59′	813
LM	Lumbini	27°29′	83°17′	100

Supplementary Table 2 | Detailed descriptions and characteristics of the snowpit sampling sites.

Snowpit ID	Glacier Name	Mountain Range	Sampling Time YYYY/MM/DD	Latitude N	Longitude E	Elevation m a.s.l	Depth cm
LH	Laohugou No.12 Glacier	Qilian	2014/05/20	39°25.668′	96°33.355′	5026	40
TG	Xiaodongkemadi Glacier	Tanggula	2014/05/17	33°04.486′	92°05.3′	5742	100
ZD	Zhadang Glacier	Nyainqêntanglha	2014/06/05	30°28.079′	90°39.041′	5800	150
YL	Yulong Glacier	Hengduan	2014/05/12	27°6.2814′	100°11.796′	4606	170
DM	Demula Glacier	Eastern Himalaya	2014/05/06	29°21.615′	97°01.060′	5404	180
QY	Qiangyong Glacier	Eastern Himalaya	2014/05/23	28°51.472′	90°13.630′	5739	155
EV	East Rongbu Glacier	Middle Himalaya	2013/05/11	28°01.852′	86°56.489′	6350	120
TH	Thorung Glacier	Middle Himalaya	2012/06/01	28°47.124′	83°55.832′	5410	42
LH-M	Laohugou No.12 Glacier	Qilian	2014/08/06	39°25.668′	96°33.355′	5026	
TG-M	Xiaodongkemadi Glacier	Tanggula	2014/07/06	33°04.486′	92°05.3′	5742	
ZD-M	Zhadang Glacier	Nyainqêntanglha	2013/08/25	30°28.08′	90°39.041′	5800	

Note: The LH-M, TG-M and ZD-M snow samples were collected during the monsoon period.

Supplementary Table 3 | WIOC/BC ratios and split times for aerosol and snowpit samples.

	Station/snowpit	WIOC ($\mu g\ cm^{-2}$)	BC ($\mu g\ cm^{-2}$)	WIOC/BC	Split time (S)
Aerosol	NM	26.23	2.28	11.5	465
	NM	35.64	3.17	11.23	468
	JS	15.11	0.76	19.91	461
	JS	15.97	1.33	11.96	458
	JS	18.43	1.33	13.84	461
	ZB	10.07	0.51	19.82	485
	ZB	28.32	2.25	12.57	479
Snowpit	DM	163.29	15.01	10.88	466
	TG	100.08	11.75	8.52	504
	ZD	41.45	6.36	6.52	467

Note: All three stations are located in remote areas with atmospheric features similar to those of the glacial regions and thus provide a good basis for comparing the aerosol and snowpit samples. Two to three aerosol samples were selected from each of the three stations. The WIOC/BC ratios of the snowpits are lower than those of aerosols for several reasons (e.g., the influence of mineral dust and BC enrichment compared to that of OC after the particles are deposited onto the glacier surface[1]).

Supplementary Table 4 | Concentrations, isotopic ($\Delta^{14}C$ and $\delta^{13}C$) compositions, ages and fossil fuel contributions of BC in the aerosol samples.

Station	Collecting date	Concentration ($\mu g\ m^{-3}$)	$\Delta^{14}C$ (‰)	Age (yr BP)	Fossil contribution (%)	$\delta^{13}C$ (‰)
NM	2013/4/1	0.12	-422.84	4,350	46.06	-25.57
NM	2013/11/1	0.07	-378.31	3,760	41.90	-25.93
NM	2014/6/1	0.1	-327.98	3,130	37.19	-23.97
NM	2014/7/1	0.08	-480.16	5,190	51.42	-25.56
LS	2013/6/29	5.05	-914.38	19,700	92.00	-24.06
LS	2013/11/6	18.31	-808.3	13,200	82.08	-24.64
LS	2014/1/5	5.6	-782.28	12,200	79.65	-23.96
LS	2013/4/1	8.44	-710.17	9,890	72.91	-24.77
NL	2013/4/1	3.71	-459.52	4,880	54.96	-24.08
NL	2013/6/2	0.59	-503.99	5,570	58.67	-24.8
NL	2013/11/14	0.66	-520.57	5,840	60.05	N.D.
DH	2013/8/7	2.15	-807.22	13,150	83.94	-25.19
DH	2013/9/12	0.99	-503.36	5,560	58.61	-25.36
DH	2014/1/11	3.27	-407.34	4,140	50.61	-24.85
KT	2013/4/8	47.52	-600.29	7,300	66.69	-26.34
KT	2013/8/7	3.49	-807.22	13,150	83.94	-25.19
KT	2013/11/10	36.13	-661.77	8,650	71.81	-25.92
KT	2014/1/10	8.72	-500.25	5,510	58.35	-25.92
ZB	2013/4/12	1.71	-158.05	1,320	29.84	-25.75
JS	2013/4/6	3.84	-218.19	1,920	34.85	-25.24
JS	2013/4/17	1.32	-410.34	4,180	50.86	N.D.
PK	2013/4/6	15.59	-170.5	1,440	30.88	-26.05
PK	2013/4/10	13.68	-265.24	2,410	38.77	N.D.
PK	2013/7/29	1.65	-692.73	9,420	74.39	-25.35
LM	2013/4/18	15.68	-300.75	2,810	41.73	N.D.
LM	2013/7/23	2.45	-498.23	5,480	58.19	-26.36
LM	2013/10/28	9.03	-359.74	3,520	46.65	-25.25
LM	2013/12/1	7.84	-407.77	4,150	50.65	-25.42

Note: Stringent natural abundance ^{14}C detection limits were met for samples collected during different seasons at four of the sites (Namco, Lhasa, Kathmandu and Lumbini). N.D. = not determined. All acronyms are the same as those used in Supplementary Table 1.

Supplementary Table 5 | BC concentrations (ng g^{-1}) in the snowpit samples collected in this study and previously reported values.

Glacier	BC (this study)	BC (previously reported)
LH	133	35[2]
TG	29	79[3]
ZD	79	114[2]
YL	34	-
DM	17	16.8[4]
QY	28	43[3]
EV	11	18[2]
TH	19	-

Note: All acronyms are the same as those used in Supplementary Table 2.

Supplementary Table 6 | Concentrations, isotopic ($\Delta^{14}C$ and $\delta^{13}C$) compositions, ages and fossil contributions of BC in the snowpit samples.

Snowpit	Concentration(ng g^{-1})	$\Delta^{14}C$(‰)	Age(yr BP)	Fossil contribution (%)	$\delta^{13}C$(‰)
LH-1	163.31	-750.41	11,100	76.67	-22.52
LH-2	64.29	-502.37	5,540	53.49	-24.22
TG-1	365.27	-314.6	2,970	35.94	-26.24
TG-2	30.83	-161.83	1,360	21.67	-25.03
DM-1	60.07	-316.92	3,000	43.08	-25.52
DM-2	178.31	-305.83	2,870	42.15	-27.34
YL-1	23.03	-348.91	3,380	45.74	-23.3
YL-2	53.1	-352.09	3,420	46.01	-25.16
QY-1	70.3	-341.8	3,300	38.49	-24.26
QY-2	13.85	-335.19	3,220	37.87	-22.96
ZD-1	11.77	-247.97	2,230	29.72	-25.7
ZD-2	10.84	-268.61	2,450	31.65	N.D.
EV	10.59	-253.81	2,290	30.26	-22.1
TH-1	26.81	-262.79	2,423	38.57	N.D.
TH-2	12.12	-450.97	4,750	54.25	-24.56
LH-M	15.32	-288.49	2,670	33.50	-27.54
TG-M	15.57	-251.21	2,260	30.02	-24.69
ZD-M	19.03	-137.45	1,130	19.39	-23.85

Note: the LH-M, TG-M and ZD-M snow samples were collected during the monsoon period, and the others are snowpit samples. All acronyms are the same as those used in Supplementary Table 2.

Supplementary Table 7 | End members of Δ^{14}C and δ^{13}C used in the BC isotopic mass-balance source apportionment calculations (mean ± SD). End members were obtained from Andersson et al. (2015)[5], whereas the Δ^{14}C and δ^{13}C values for C3 plants were derived from the BC levels of aerosols emitted from yak dung combustion.

	C3 plant	Liquid fossil fuel	Coal
^{14}C	70	-1000	-1000
δ^{13}C	-26.7±1.8	-25.5±1.3	-23.38±1.3

Supplementary References

1. Xu B, Cao J, Joswiak DR, Liu X, Zhao H, He J. Post-depositional enrichment of black soot in snow-pack and accelerated melting of Tibetan glaciers. *Environmental Research Letters* **7**, 014022 (2012).

2. Ming J*, et al.* Black Carbon (BC) in the snow of glaciers in west China and its potential effects on albedos. *Atmospheric Research* **92**, 114-123 (2009).

3. Xu B-Q*, et al.* Deposition of anthropogenic aerosols in a southeastern Tibetan glacier. *Journal of Geophysical Research-Atmospheres* **114**, (2009).

4. Xu B*, et al.* Black soot and the survival of Tibetan glaciers. *Proceedings of the National Academy of Sciences* **106**, 22114-22118 (2009).

5. Andersson A*, et al.* Regionally-Varying Combustion Sources of the January 2013 Severe Haze Events over Eastern China. *Environmental science & technology* **49**, 2038-2043 (2015).
